# Echocardiographic estimation of pulmonary arterial and right atrial pressures in children with congenital heart disease: a comprehensive prospective study and introduction of novel equations

**DOI:** 10.1186/s44348-024-00023-4

**Published:** 2024-08-08

**Authors:** Elaheh Malakan Rad, Reza Elhamian, Keyhan Sayadpour Zanjani, Reza Shabanian, Ehsan Aghaei Moghadam, Mohamad Taghi Majnoon, Aliakbar Zeinaloo

**Affiliations:** 1 Department of Pediatric Cardiology, Children’s Medical Center (Pediatric Center of Excellence), Tehran University of Medical Sciences, Tehran, Iran; 2 Fetal and Pediatric Cardiovascular Research Center, Children’s Medical Center (Pediatric Center of Excellence), Tehran University of Medical Sciences, Tehran, Iran; 3 Hakim Children’s Hospital, Tehran University of Medical Sciences, Tehran, Iran

**Keywords:** Pulmonary arterial pressure, Echocardiography, Right atrial pressure, Bland–Altman analysis, Congenital heart disease, Receiver operating characteristic curve, Children

## Abstract

**Background:**

Pediatric pulmonary hypertension (PH) is characterized by a mean pulmonary arterial pressure exceeding 20 mmHg. There is limited research on the suitability of adult-based methods for estimating PH in pediatric populations. Using established formulas for adults, this study aimed to evaluate the correlation between echocardiographic estimates of systolic, diastolic, and mean pulmonary arterial pressures, and mean right atrial pressures in children with congenital heart disease (CHD).

**Methods:**

A prospective study was conducted involving children with CHD undergoing cardiac catheterization without prior cardiac surgery. We used echocardiography to estimate pulmonary and right atrial pressures and compared these with invasively measured values. Four reliable regression equations were developed to estimate systolic, diastolic, and mean pulmonary arterial pressures, and mean right atrial pressures. Cutoff values were determined to predict the occurrence of PH. Linear regression, Bland–Altman analysis, and receiver operating characteristic curve analysis were performed to assess the accuracy of echocardiography and establish diagnostic thresholds for PH.

**Results:**

The study involved 55 children (23 with normal pulmonary arterial pressure and 32 with PH) with acyanotic CHD aged 1 to 192 months. Four equations were developed to detect high pulmonary arterial pressures, with cutoff values of 32.9 for systolic pulmonary arterial pressure, 14.95 for diastolic pulmonary arterial pressure, and 20.7 for mean pulmonary arterial pressure. The results showed high sensitivity and moderate specificity but a tendency to underestimate systolic and mean pulmonary arterial pressures at higher pressures.

**Conclusions:**

The study provides valuable insights into the use of adult-based echocardiographic formulas for estimating PH in pediatric patients with acyanotic CHD.

**Supplementary Information:**

The online version contains supplementary material available at 10.1186/s44348-024-00023-4.

## Background

Pediatric pulmonary hypertension (PH), defined as a mean pulmonary arterial pressure (MPAP) of more than 20 mmHg, can be associated with significant morbidity and mortality. Therefore, early diagnosis is of critical importance [1, 2]. Right heart catheterization is the gold standard for the diagnosis of PH. Its invasiveness and complexity, however, limit its application. As a result, there has been a continuing quest to find reliable echocardiographic surrogate methods for diagnosing PH and estimating pulmonary arterial pressure [3–6]. Although there are several methods for estimating and diagnosing PH in adults, few studies have investigated the applicability of these methods in children [7]. The aims of this study are threefold: to investigate the correlation between values obtained by application of the adults' formula for estimation of systolic pulmonary arterial pressure (SPAP), diastolic pulmonary arterial pressure (DPAP), and MPAP, and invasively measured counterparts; to study whether the dichotomous variables such as pulmonary artery acceleration time (PAAT) of fewer than 90 ms, tricuspid systolic velocity < 12 cm/sec, right ventricular isovolumic relaxation time > 75 ms, and right ventricular outflow tract acceleration time of < 100 ms can predict the presence of PH; and finally to investigate whether the recommendations of the American Society of Echocardiography (ASE) regarding estimation of mean right atrial pressure (MRAP) in the adults can reliably predict MRAP in the pediatric population [8, 9].

## Methods

### Study design

A prospective study was conducted in the Children’s Medical Center (Tehran, Iran) from March 2020 to March 2021.

### Study population

We included children admitted to the Children's Medical Center aged over 30 days and under 16 years, in normal sinus rhythm, requiring diagnostic or interventional cardiac catheterization without prior cardiac surgery. Exclusion criteria were as follows: complex congenital heart disease (CHD), right-to-left shunt, ventricular dysfunction, arrhythmia, noncardiac causes of PH, scimitar syndrome, inferior vena cava (IVC) obstruction, specific conditions during an echocardiographic examination or cardiac catheterization (oxygen saturation < 94%, hypotension or hypertension, bradycardia or tachycardia, poor acoustic window), and lack of informed consent. Demographic, echocardiographic, and cardiac catheterization data were collected.

### Sample size

We used G*Power (Heinrich Heine University Düsseldorf) to calculate the sample size (one sample case, two-tails, effect size = 0.5, α error probability = 0.05, power = 0.95) [10].

### Echocardiographic examination

A comprehensive echocardiographic examination was conducted within 24 h of cardiac catheterization in all patients using various techniques (Affiniti 50 C Echocardiography Machine, Philips). Probes S4-2 and S8-3 were chosen based on patient size. A total of 29 echocardiographic parameters, outlined in Supplementary Table 1 (Additional file 1), were measured as previously described [11, 12]. The average value of each echocardiographic parameter was calculated over three consecutive cycles. An expert pediatric cardiologist performed all echocardiographic examinations.

Estimated SPAP, DPAP, and MPAP were calculated using the 18 popular formulas in the literature (Table 1) [1, 4–8, 13–29].

**Table 1:** Established formulas for echocardiographic estimation of SPAP, DPAP, MPAP, and MRAP, as well as the correlation between these formulas and measured pressures in 55 children with congenital heart disease

Established formula	Correlation between echocardiographically estimated and measured pulmonary arterial pressure by cardiac catheterization with derived regression equations for predicting pulmonary arterial pressure				
	R^2^	Adjusted R^2^	Correlation coefficient	*P*-value	Regression equation
Existing formula for ESPAP					
ESPAP1 = 4 × (TR peak velocity)^2^	0.60	0.59	0.77	< 0.001	SPAP1 = 0.45 × ESPAP1 + 18.88
ESPAP2 = 4 × (TR peak velocity)^2^ + estimated RAP	0.58	0.58	0.77	< 0.001	SPAP2 = 0.44 × ESPAP2 + 17.2
ESPAP3 = TR peak pressure gradient	0.59	0.58	0.77	< 0.001	SPAP3 = 0.45 × ESPAP3 + 18.27
ESPAP4 = TR peak pressure gradient + estimated RAP	0.57	0.56	0.78	< 0.001	SPAP4 = 16.72 + 0.44 × ESPAP4
Existing formula for EDPAP					
EDPAP1 = 4 × (PR end-diastolic velocity)^2^	0.37	0.36	0.61	< 0.001	12.73 + 0.89 × EDPAP1
EDPAP2 = 4 × (PR end-diastolic velocity)^2^ + estimated RAP	0.25	0.24	0.50	< 0.001	10.41 + 0.68 × EDPAP2
Existing formula for EMPAP					
EMPAP1 = (0.61 × TR peak pressure gradient) + 2	0.63	0.62	0.79	< 0.001	12.68 + 0.5 × EMPAP1
EMPAP2 = {0.61 × (TR peak pressure gradient + estimated RAP)} + 2	0.60	0.59	0.78	< 0.001	11.71 + 0.49 × EMPAP2
EMPAP3 = 90 – (0.62 × RVOT AT)	0.01	–0.01	0.01	0.475	-
EMPAP4 = 79 – (0.45 × RVOT AT)	0.01	–0.01	0.01	0.475	-
EMPAP5 = TR mean pressure gradient + RAP	0.55	0.54	0.74	< 0.001	12.98 + 0.49 × EMPAP5
EMPAP6 = (PR peak pressure gradient + 2) / 0.7	0.34	0.33	0.59	< 0.001	16.5 + 0.33 × EMPAP6
EMPAP7 = 4 × (PR peak velocity^a^)^2^ + estimated RAP	0.29	0.28	0.54	< 0.001	15.32 + 1.14 × EMPAP7
EMPAP8 = 80 – (0.5 × RVOT AT)	0.01	–0.01	0.01	0.475	-
EMPAP9 = {0.60 × (TR peak pressure gradient + estimated RAP)} + 2.1	0.60	0.59	0.78	< 0.001	11.64 + 0.5 × EMPAP9
EMPAP10 = 83 – {148 (RVOT AT / RVOT ET)}	0.07	0.05	0.26	0.061	-
EMPAP11 = 0.61 × TR peak pressure gradient + 1.95 mmHg	0.60	0.59	0.78	< 0.001	11.73 + 0.49 × EMPAP11
EMPAP12 = 48 – (0.28 × pulmonary artery AT)	0.27	0.08	0.06	0.040	15.4 + 0.44 × EMPAP12

All velocities are in meters per second (velocities used in the formulas mentioned above were converted from centimeters per second to meters per second before inserting them in the formulas). All "time" variables are in milliseconds. All "pressure" variables are in mmHg

*SPAP* Systolic pulmonary arterial pressure, *DPAP* Diastolic pulmonary arterial pressure, *MPAP* Mean pulmonary arterial pressure, *MRAP* mean right atrial pressure, *ESPAP* Estimated systolic pulmonary arterial pressure, *TR* Tricuspid regurgitation, *RAP* Right atrial pressure, *EDPAP* Estimated diastolic pulmonary arterial pressure, *PR* Pulmonary regurgitation, *EMPAP* Estimated mean pulmonary arterial pressure, *RVOT* Right ventricular outflow tract, *AT* Acceleration time, *ET* Ejection time

^a^The PR jet velocity was measured at the beginning of diastole

Right atrial pressure (RAP) estimation was followed by modification of the guidelines recommended by the ASE [3]. The reason for this was threefold: firstly, ASE guidelines were developed for the adult population; secondly, in our study population, even the patient with an invasively measured MRAP of 20 mmHg did not have an IVC diameter exceeding 2.1 cm (see Supplementary Fig. 1 in Additional file 1). Thirdly, as of the present, there are no established guidelines specifically designed for infants and children. Hence, cases were deemed normal RAP if the IVC diameter measured less than 2.1 cm and demonstrated more than 50% reduction during inspiration, resulting in an estimated mean pressure of 3 mmHg. Children who exhibited an inspiratory IVC collapse of less than 50% were classified as having an estimated mean atrial pressure of 8 mmHg [2]. The IVC, right ventricle, and pulmonary arterial dimensions were measured as described earlier [3].

We sedated infants and children who were not completely calm using intranasal or oral midazolam at a dose of 0.2 to 0.3 mg/kg, a maximum of 10 mg, or oral chloral hydrate at a dose of 25 to 50 mg/kg in children (a maximum dose of 500 mg).

### Cardiac catheterization

Patients had cardiac catheterization within 24 h of the echocardiographic examination. They were under general anesthesia with sevoflurane and mechanically ventilated with 21% oxygen. Heart rate, oxygen saturation, end-tidal carbon dioxide, and blood pressure were continuously monitored. PH was defined as MPAP exceeding 20 mmHg, and RAP of 3 to 6 mmHg was considered normal and ≥ 7 mmHg as increased [1, 30]. SPAP exceeding 36 mmHg indicated elevated SPAP; DPAP exceeding 21 mmHg indicated elevated DPAP [21, 31].

### Dichotomous variables for prediction of PH

We examined the relationship between the presence or absence of specific dichotomous variables and the presence or absence of PH, defined as a MPAP greater than 20 mmHg [4, 6, 24, 32–34]. The dichotomous variables under investigation include the following: tricuspid systolic velocity less than 12 cm/sec; right ventricular isovolumic relaxation time greater than 75 ms; acceleration time of the right ventricular outflow tract less than 100 ms; PAAT less than 90 and 60 ms; PAAT to right ventricular ejection time (RVET) ratio less than 0.31, 0.29, 0.25, and 0.23; tricuspid annular plane systolic excursion less than 16 mm; Tei index greater than 0.36; the ratio of right ventricular basal diameter to left ventricular basal diameter greater than 1; PA to aortic size greater than 1.5 and 2; PAAT to aortic acceleration time ratio less than or equal to 1 and 0.7; and the presence of a mid-systolic notch in the Doppler of the right ventricular outflow tract.

### Statistical analysis

We used the Shapiro–Wilk test to assess data distribution normality. Mean ± standard deviation and range of continuous data are presented. Due to the small sample size and multicollinearity, we used univariate linear regression to examine the link between echocardiographically estimated pulmonary pressures using the established formulas in adults and invasively measured parameters. Pearson chi-squared test and Fisher exact test (if the expected number < 5) were used to analyze the correlation of dichotomous variables with the presence or absence of PH. Bland–Altman plots assessed agreement between invasively measured and estimated pulmonary arterial pressures. receiver operating characteristic curves were constructed to assess the regression equations' accuracy for estimating pulmonary arterial pressures. These curves were used to determine optimal cutoff values for predicting systolic, diastolic, and mean pulmonary arterial hypertension, ensuring an appropriate balance between sensitivity and specificity. For our study, we established cutoff values for each regression equation that estimates SPAP, DPAP, and MPAP. These cutoff values determine the point at which a patient's condition is classified as either normal or indicative of systolic, diastolic, or mean pulmonary arterial hypertension. This approach facilitates the categorization of patient conditions based on their measured values against these predefined thresholds.

The statistical methodology to formulate the proposed novel formulas involved selecting variables from existing formulas with the highest R^2^ and β coefficients (correlation coefficients). This approach aimed to create a multivariable equation demonstrating a stronger correlation between invasively measured and echocardiographically estimated diastolic and mean pulmonary arterial pressures.

R^2^ is a statistical metric that quantifies how much of the variance in a dependent variable can be explained by one or more independent variables in a regression model. For instance, an R^2^ value of 0.70 suggests that the regression model accounts for 70% of the observed variability in the target variable. A higher R^2^ value generally indicates a greater capacity of the model to elucidate variability. Traditionally, an R^2^ value exceeding 0.7 indicates a substantial effect size. Conversely, the β coefficient measures the degree of change in the outcome variable for each unit of change in the predictor variable. Beta coefficient values range from –1 to + 1. Values between 0 and 0.19 are characterized as very weak, 0.20 to 0.39 as weak, 0.40 to 0.59 as moderate, 0.60 to 0.79 as strong, and 0.80 to 1 as extremely strong. A *P*-value of < 0.05 was considered statistically significant.

### Ethics statement

This study was approved by the Institutional Research Ethics Committee of Children's Medical Center affiliated with Tehran University of Medical Sciences (No. IR.TUMS.CHMC.REC.1400.280). Informed consent was obtained from the patients' parents.

The study was conducted according to the Declaration of Helsinki on Medical Research Involving Human Subjects [35].

## Results

### Baseline patients’ characteristics

A cohort of 55 pediatric patients with CHD was examined, consisting of 28 male and 27 female patients. Of these, 84% exhibited a lesion characterized by a left-to-right shunt. Within this population, 23 patients (42%) presented with normal pulmonary arterial pressure, while the remaining 32 patients (58%) were diagnosed with PH. The patients' diagnoses included patent ductus arteriosus (22 cases), ventricular septal defect (14 cases), atrial septal defect (7 cases), primary PH (2 cases), aortic stenosis (2 cases), atrioventricular septal defect (2 cases), and single cases each of congenital mitral stenosis, left PA stenosis, subaortic web, arterial tortuosity syndrome, coarctation of the aorta, and patent foramen ovale. Table 2 provides an overview of the study population's basic characteristics and descriptive echocardiographic statistics.

**Table 2:** The summary of the study's population basic and echocardiographic descriptive statistics

Variable	**Minimum**	**Maximum**	**Mean**	**Standard deviation**
Age (mo)	1.00	192.00	46.47	45.14
Aortic acceleration time (msec)	50.00	165.00	84.31	22.39
Aortic size (mm)	9.20	24.90	14.55	2.95
Inferior vena cava				
Collapsibility index	0.29	0.78	0.54	0.12
Minimal diameter (cm)	0.09	1.07	0.35	0.19
Maximal diameter (cm)	0.21	1.60	0.75	0.30
Left ventricular dimension (mm)	16.50	48.60	29.51	6.43
Mitral valve E velocity (cm/sec)	3.19	269.00	119.52	45.42
Mitral valve Em velocity (cm/sec)	5.60	42.00	17.58	6.30
PAAT (msec)	58.00	178.00	98.84	23.43
Pulmonary artery deceleration time (msec)	34.00	372.00	197.69	60.53
PAAT to pulmonary artery deceleration time	0.24	2.44	0.57	0.33
Pulmonary artery diastolic pressure (mmHg)	6.00	45.00	15.78	6.64
Pulmonary artery ejection time (msec)	117.00	483.00	297.79	66.21
Pulmonary artery mean pressure (mmHg)	11.00	73.00	24.33	10.38
Pulmonary artery size (mm)	8.70	31.00	16.61	4.21
Pulmonary artery systolic pressure (mmHg)	17.00	101.00	33.93	15.76
PR – end velocity (cm/sec)	21.00	259.00	81.49	45.03
PR – peak (early) velocity (cm/sec)	70.00	360.00	174.25	76.81
PR – peak pressure gradient (mmHg)	2.00	52.00	14.48	12.79
Right atrial mean pressure measured by cardiac catheterization (mmHg)	4.00	20.00	9.41	3.04
Right ventricular IVCT (msec)	33.00	86.00	56.36	13.70
Right ventricular IVRT (msec)	23.00	90.00	50.63	14.35
Right ventricular IVRT/IVCT	0.41	1.72	0.93	0.3
Right ventricular outflow tract acceleration time (msec)	72.00	444.00	161.42	75.74
Right ventricular outflow tract velocity time integral (cm)	4.80	43.80	16.79	7.59
Right ventricular basal dimension (mm)	15.70	40.20	25.66	5.94
Right ventricular mid-cavity dimension (mm)	10.90	39.80	21.81	6.93
Right ventricular size-3 (mm)	26.10	72.00	42.79	9.00
Tricuspid annular plane systolic excursion (mm)	8.00	29.00	18.49	4.31
TR mean pressure gradient (mmHg)	2.00	86.00	18.60	15.48
TR peak pressure gradient (mmHg)	4.00	179.00	34.68	26.78
TR peak velocity (m/sec)	0.69	66.90	2.74	0.99
TR velocity time integral (centimeter)	14.10	190.00	58.02	33.87
Tricuspid valve E velocity (cm/sec)	46.00	388.00	98.63	52.58
Tricuspid valve Em velocity (cm/sec)	10.30	44.00	19.46	6.16
Tricuspid valve Sm velocity (cm/sec)	8.00	22.00	13.23	2.95
Tricuspid valve Sm velocity time integral (cm/sec)	0.87	3.50	2.36	0.59

*PAAT* Pulmonary artery acceleration time, *PR* Pulmonary regurgitation, *IVCT* Isovolumic contraction time, *IVRT* Isovolumic relaxation time, *TR* Tricuspid regurgitation

### Echocardiographic estimation of SPAP

Among the four formulas presented in Table 1, the first formula, “4×(tricuspid regurgitation [TR] peak velocity)^2^” and fourth formula, “TR peak pressure gradient + estimated RAP,” which integrated TR peak velocity and peak pressure gradient, exhibited the strongest correlation with invasively measured SPAP (r = 0.77, *P* < 0.001 and r = 0.48, *P* < 0.001, respectively).

### Echocardiographic estimation of DPAP

Both of the formulas presented in Table 1 for estimating DPAP “4 × (pulmonary regurgitation [PR] end-diastolic velocity)^2”^ and “4 × (PR end-diastolic velocity)^2^ + estimated RAP” displayed a significant but weak correlation with the values obtained by cardiac catheterization. However, the inclusion of echocardiographically estimated RAP in the formula reduced the strength of the correlation coefficient (r = 0.37, *P* < 0.001 compared to r = 0.25, *P* < 0.001).

### Echocardiographic estimation of MPAP

Of the 12 established formulas shown in Table 1 for estimating MPAP, we found a significant and strong correlation between echocardiographically estimated MPAP and invasively measured pulmonary arterial pressure by applying formulas 1, 2, 5, 6, 7, and 9. However, there was no significant correlation between echocardiographically estimated MPAP and invasively measured pulmonary arterial pressure using formulas 3, 4, 8, 10, 11, and 12 (Table 1).

### Echocardiographic prediction of pulmonary arterial hypertension using dichotomous variables

No statistically significant relationship was found between the presence of any dichotomous variables on echocardiography and pulmonary arterial hypertension at cardiac catheterization (Table 3).

**Table 3:** Association between predictive dichotomous variables for identifying PH and invasively measured mean pulmonary arterial pressure in 55 children with congenital heart disease

No.	**Variable**	**Reference**	***P*-value (two-sided)**	
			Pearson chi-square test	Fisher exact test
1	Tricuspid Sm velocity < 12 cm/sec	Reference no. 4	0.68	-
2	Right ventricular isovolumic relaxation time > 75 ms	Reference no. 4	-	1.00
3	Acceleration time of right ventricular outflow tract < 100 ms	Reference no. 4	-	0.17
4	Mid-systolic notch in the Doppler of right ventricular outflow tract	Reference no. 4	0.32	-
5	The ratio of right ventricular basal diameter to left ventricular basal diameter > 1	Reference no. 6	0.02	-
	PAAT (msec)			
6	< 90	Reference no. 8	-	1.00
7	< 60	A	-	1.00
	PAAT/RVET			
8	< 0.31	Reference no. 8	0.28	-
9	< 0.29	B	-	0.19
10	< 0.25		-	0.21
11	< 0.23		-	0.10
12	Tricuspid annular plane systolic excursion < 16 mm	Reference no. 3	0.39	-
	Tei index			
13	> 0.36	Reference no. 34	0.88	-
14	> 0.80	C	-	0.26
	Pulmonary artery to aortic size			
15	> 1.5	D	-	0.26
16	> 2		-	1.00
17	PAAT to pulmonary artery deceleration time < 0.3	E	-	0.06
	PAAT to aortic acceleration time			
18	≤ 1		0.35	-
19	≤ 0.7		-	0.50

*PH* Pulmonary hypertension, *PAAT* Pulmonary artery acceleration time, *RVET* Right ventricular ejection time

A, given that a value of 90 did not yield statistical significance, the authors designed this figure to assess whether a lower value might achieve such significance. B, given that a value of 0.31 did not yield statistical significance, the authors designed this figure to assess whether a lower value might achieve such significance. C, given that a value of 0.36 did not yield statistical significance, the authors almost doubled this figure to assess whether a higher value might achieve such significance. D, this novel index, developed by the authors, aims to explore the relationship between the dilation of the main pulmonary artery and the occurrence of PH. E, we explored three new ratios after finding no significant correlation between the absolute values of PAAT and PH in children. We aimed to determine if the ratio of PAAT to other relevant time variables could assist in predicting PH. We focused on analyzing extreme values to enhance the likelihood of uncovering existing relationships

### Novel formulas for estimation of DPAP and MPAP

Taking into account the R^2^ and correlation coefficient values obtained from the estimated SPAP formula 1 (ESPAP1), estimated DPAP formula 1 (EDPAP1), and estimated MPAP formula 1 (EMPAP1), we calculated the MPAP and DPAP as follows (these three formulas are delineated in Table 1):

- $$\text{MPAP }\left(\text{mmHg}\right)=\frac{\text{SPAP}+\left(2\times \text{DPAP}\right)}{3}$$  
- $$\text{MPAP }\left(\text{mmHg}\right)=\frac{\text{ESPAP}1+\left(2\times \text{EDPAP}1\right)}{3}$$

The following new multiparametric formula for EMPAP was derived:

$$\text{EMPAP}=\frac{\left\{4\times {\left(\text{TR peak velocity}\right)}^{2}\right\}+\left\{2\times {\left(4\times \text{PR end}-\text{diastolic velocity}\right)}^{2}\right\}}{3}$$

- $$\text{DPAP }\left(\text{mmHg}\right)=\frac{3\times \text{MPAP}-\text{SPAP}}{2}$$  
- $$\text{DPAP }\left(\text{mmHg}\right)=\frac{3\times \text{EMPAP}1-\text{ESPAP}1}{2}$$

The following new multiparametric formula for EDPAP was derived:

$$\text{EDPAP}=\frac{3\times \left\{0.61\times \left(\text{TR peak pressure gradient}\right)+2\right\}-\left\{4\times {\left(\text{TR peak velocity}\right)}^{2}\right\}}{2}$$

After applying these formulas, the R^2^ value and correlation coefficient values of the EDPAP and EMPAP formulas increased compared to the DPAP formula 1 and MPAP formula 1 in Table 1. Specifically, the R^2^ value and correlation coefficient for DPAP improved from 0.37 and 0.61 to 0.49 and 0.70, respectively. Similarly, for MPAP, these metrics showed an enhancement, rising from 0.63 and 0.79 to 0.65 and 0.80, respectively as detailed in Table 4.

**Table 4:** Four reliable regression equations to estimate SPAP, DPAP, MPAP, and MRAP in 55 children with congenital heart disease, as well as cutoff values, sensitivity, and specificity for predicting elevated SPAP, DPAP, and MPAP

Eachocardiographically estimated pressure	Linear regression				ROC curve					
	Regression equation	Correlation coefficient	R^2^	*P*-value	AUC	95% CI	*P*-value	Cutoff value	Sensitivity (%)	Specificity (%)
SPAP	= 0.45 × ESPAP1 + 18.88	0.78	0.57	< 0.001	0.92	0.84–1.00	< 0.001	32.9	94.1	78.4
DPAP	= 8.4 + 0.41 × new EDPAP	0.70	0.49	< 0.001	0.88	0.74–1.00	0.006	14.95	100	62.0
MPAP	= 14.45 + 0.73 × new EMPAP	0.80	0.65	< 0.001	0.78	0.66–0.90	< 0.001	20.7	80.6	58.3
MRAP	= 5.51 + 5.75 × BSA-indexed IVC_min_	0.67	0.45	< 0.001	-	-	-	-	-	-

*SPAP* systolic pulmonary arterial pressure, *DPAP* diastolic pulmonary arterial pressure, *MPAP* Mean pulmonary arterial pressure, *MRAP* mean right atrial pressure, *ROC* receiver operating characteristic, *AUC* area under the curve, *CI* confidence interval, *ESPAP* estimated systolic pulmonary arterial pressure, *EDPAP* estimated diastolic pulmonary arterial pressure, *EMPAP* Estimated mean pulmonary arterial pressure, *BSA* Body surface area, *IVC*_*min*_ minimal inferior vena cava

### Echocardiographic estimation of RAP

Using Fisher exact test, we compared groups with normal and high MRAP, categorizing them based on echo-estimated and catheter-measured values. Only in 11 out of 29 patients (approximately 40%), the echocardiographic and cardiac catheterization categories matched. Nevertheless, among the patients identified with high RAP via cardiac catheterization, 16 patients (55%) were erroneously estimated as normal by echocardiography. Conversely, only one patient with a normal RAP measurement from cardiac catheterization was inaccurately assessed as high by echocardiography. Additionally, the cross-tabulation analysis showed a nonsignificant *P*-value (*P* = 1).

The correlation between the IVC collapsibility index (IVCCI) and MRAP, as measured by cardiac catheterization, was statistically insignificant (*P* = 0.25). Similarly, the correlation between maximal IVC diameter and MRAP was statistically insignificant (*P* = 0.07).

A linear regression analysis between IVC size and invasively measured MRAP showed a significant correlation with minimal IVC size, as well as minimal and maximal IVC dimensions indexed by body surface area (BSA).

Based on the minimal IVC diameter (in cm), the regression equation for predicting the MRAP was as follows: predicted MRAP (mmHg) = 5.98 + 7.17 × minimal IVC diameter (r = 0.43, *P* = 0.022). However, the most robust correlation was between RAP and BSA-indexed minimal IVC size (r = 0.67, *P* < 0.001), as shown in the following equation (Table 5): predicted MRAP (mmHg) = 5.51 + 5.75 × BSA-indexed minimal IVC diameter.

**Table 5:** Correlation between minimal and maximal size of IVC and mean right atrial pressure measured by cardiac catheterization

Variable	**R**^2^	**Adjusted** R^2^	Correlation c**oefficient**	***P***-**value**	**Regression equation**
**IVC**_**min**_** (cm)**	0.18	0.15	0.43	0.022	Predicted MRAP = 5.98 × IVC_min_ + 7.17
**IVC**_**max**_** (cm)**	0.12	0.08	0.34	0.070	Predicted MRAP = 3.422 × IVC_max_ + 6.774
**BSA-indexed IVC**_**min**_	0.45	0.43	0.67	< 0.001	Predicted MRAP = 5.75 × BSA-indexed IVC_min_ + 5.51
**BSA-indexed IVC**_**max**_	0.27	0.24	0.52	0.004	Predicted MRAP = 2.856 × indexed IVC_max_ + 5.281
**IVC**CI^a^	0.05	0.01	0.22	0.250	-
**BSA-**indexed **IVC**CI	0.05	0.01	0.22	0.250	-

*IVC*_*min*_ minimal inferior vena cava, *MRAP* mean right atrial pressure, *IVC*_*max*_ maximal inferior vena cava, *BSA* body surface area, *IVCCI* inferior vena cava collapsibility index

^a^ $$\text{IVCC}=\frac{{\text{IVC}}_{\text{max}}-{\text{IVC}}_{\text{min}}}{{\text{IVC}}_{\text{max}}}\times 100$$

Table 4 lists the most reliable regression equations for estimating SPAP, DPAP, MPAP, and MRAP in 55 children with acyanotic CHD.

### Agreement between invasively measured SPAP, DPAP, MPAP, and MRAP, and the estimated values (Bland–Altman plots)

**Fig. 1:**
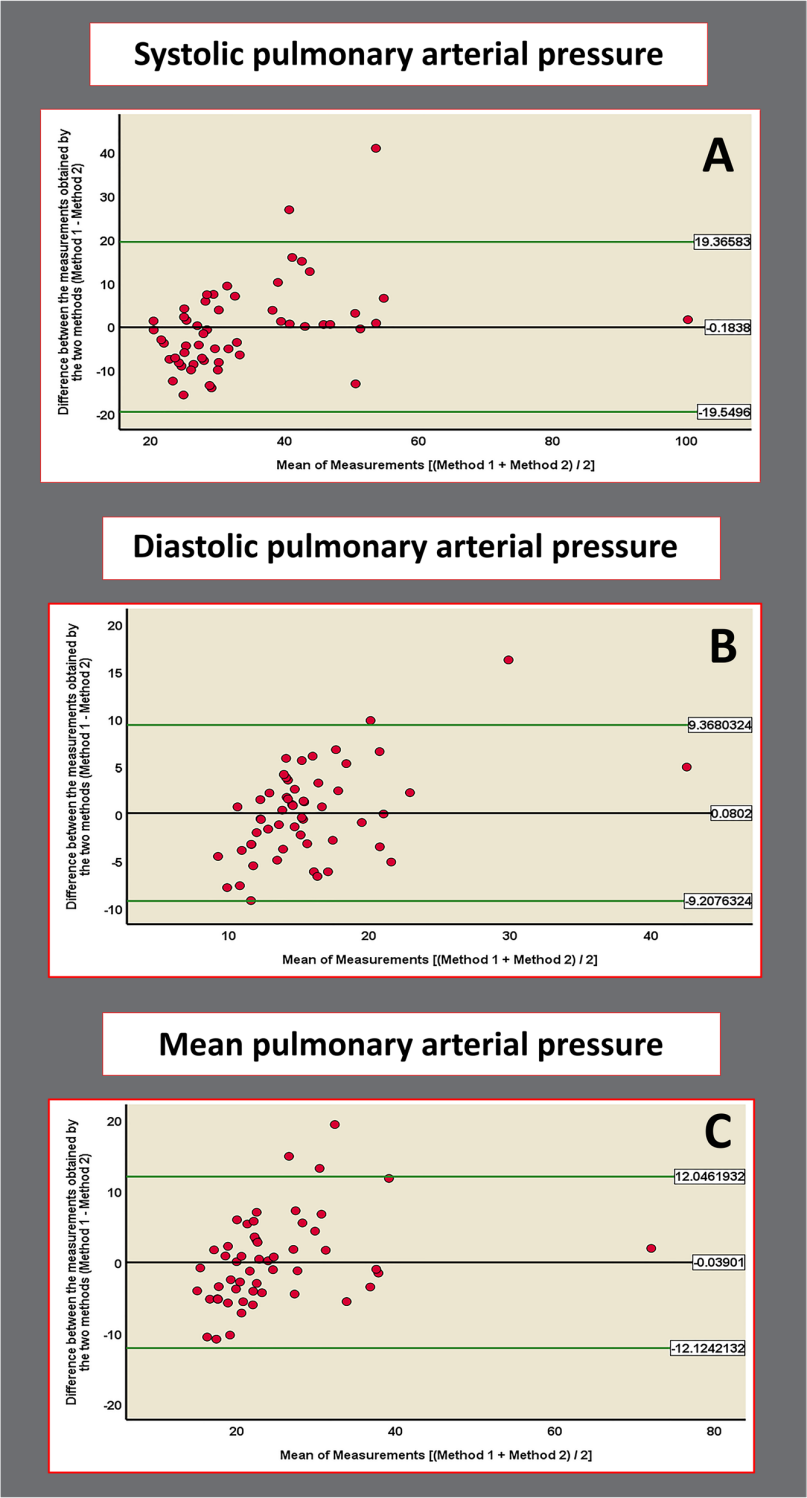
Bland–Altman plots for (**A**) systolic pulmonary arterial pressure, (**B**) diastolic pulmonary arterial pressure, and (**C**) mean pulmonary arterial pressure. The y-axis represents the variation between measured and calculated pressures (method 1, cardiac catheterization; method 2, echocardiography), while the x-axis shows the mean of the readings for each patient. The line y = 0 represents perfect agreement. **A** Points cluster near zero, with < 5% outside the upper/lower 95% confidence intervals. However, there is a trend of points shifting from below to above the mean, indicating a size-related or proportional bias. **B** No mean offset, < 5% of points outside the confidence interval, but tighter clustering on the right side. (**C**) Similar to (**A**), 5% of points outside the confidence interval, with tighter clustering on the right side. **A** and **B** show a distinct trend as we progress from left to right along the plots, corresponding to a shift from lower mean measurement values to higher ones. Specifically, there is an increasing number of data points located above the center line in this direction. This pattern strongly suggests the presence of proportional bias, which could be indicative of the overestimation of method 1 or, more accurately, the underestimation of method 2. Since method 1 involves values obtained through cardiac catheterization, it cannot reasonably be considered to be overestimated

The Bland–Altman plots were utilized to evaluate the agreement between invasively measured SPAP, DPAP, MPAP, MRAP, and MRAP, and the corresponding estimated values derived from the previously mentioned equations. The mean difference ± standard deviation between the invasively measured pressures and the estimated values using the mentioned equations were as follows: SPAP, –0.18 ± 9.97 mmHg (95% confidence interval [CI] of difference, –2.88 to 2.51; *P* = 0.892); DPAP, 0.08 ± 4.74 mmHg (95% CI of difference, –1.20 to 1.36; *P* = 0.901); MPAP, –0.04 ± 6.17 (95% CI of difference, –1.71 to 1.63; *P* = 0.963); and MRAP, –0.0001 ± 2.26 mmHg (95% CI of difference, –0.86 to 0.86; *P* = 1.000) (Fig. 1).

### Cutoff values for prediction of PH

**Fig. 2:**
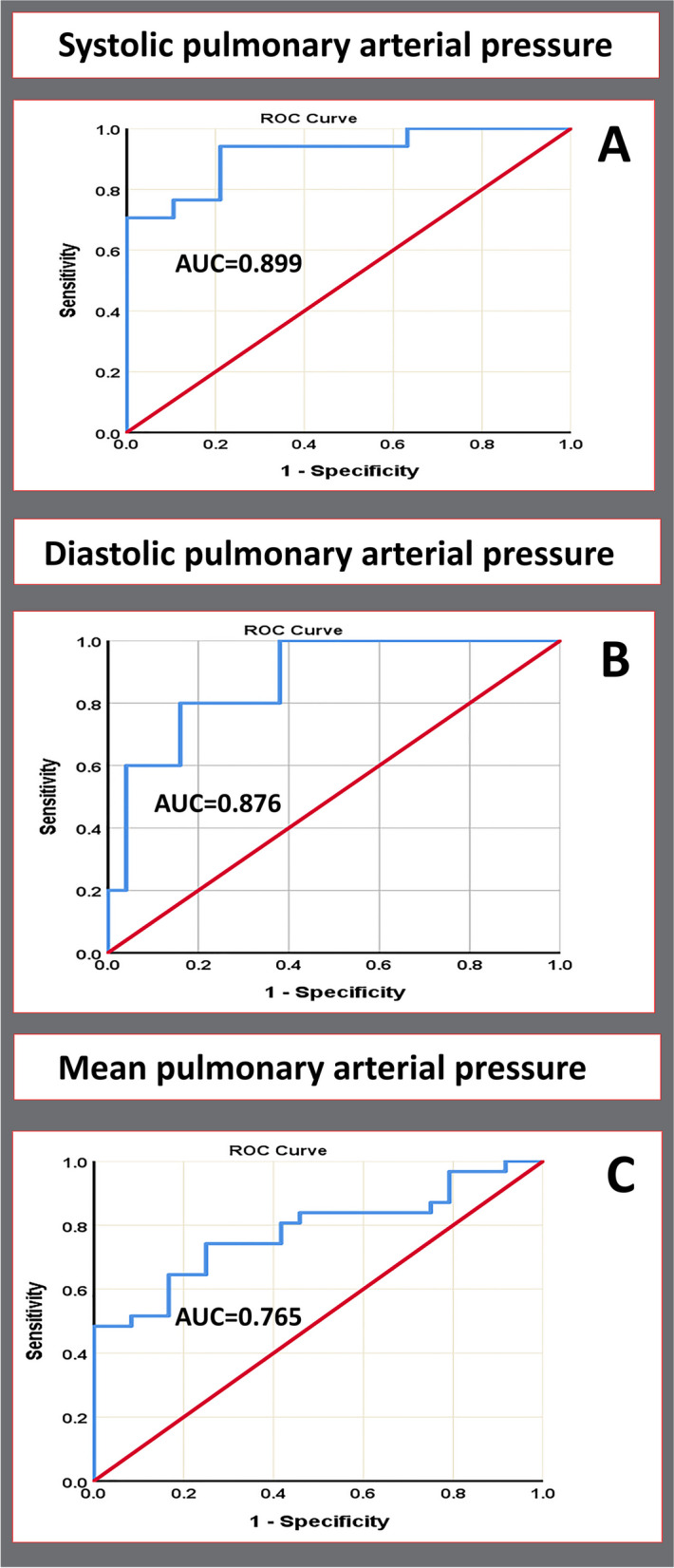
Receiver operating characteristic analyses in 55 children with congenital heart disease for (**A**) systolic pulmonary arterial pressure, (**B**) diastolic pulmonary arterial pressure, and (**C**) mean pulmonary arterial pressure, with an area under the curve (AUC) of 0.920, 0.876, and 0.780, respectively

The cutoff values for estimating SPAP, DPAP, and MPAP echocardiographically, as derived from the regression equations in Table 4, are as follows: 32.9 for SPAP (with an area under the curve [AUC] of 0.92, 94.1% sensitivity, and 78.4% specificity), 14.95 for DPAP (AUC of 0.88, 100% sensitivity, 62% specificity), and 20.7 for MPAP (AUC of 0.78, 80.6% sensitivity, 58.3% specificity) (Table 4, Fig. 2).

## Discussion

### Most robust equations for the prediction of SPAP, DPAP, MPAP and MRAP in children with acyanotic CHD

We presented the most reliable equations for echocardiographic estimation of SPAP, DPAP, MPAP, and MRAP in a cohort of 55 infants and young children with CHD. Our results indicate that values obtained from echocardiography are generally congruent with invasively measured values at lower pressure levels. However, it is noteworthy that there is a tendency to underestimate SPAP and, to a lesser extent, MPAP at higher pressure levels.

Additionally, we supplied cutoff values with acceptable sensitivity and specificity for the echocardiographic prediction of elevated PA pressure. The established cutoff values possess significant clinical utility and serve a multitude of purposes, including screening for PH, early detection of the condition, monitoring treatment efficacy, assessing risk profiles, informing the development of tailored treatment plans, offering prognostic insights, minimizing the necessity for invasive procedures, and facilitating ongoing research endeavors.

This study provided several crucial insights into how to extrapolate our understanding of echocardiographic estimation of pulmonary arterial pressure in adults to young children.

### Most reliable existing adult formulas for application in children

In this study involving a cohort of 55 pediatric patients with acyanotic CHD, it was observed that for estimating SPAP, all four formulas listed in Table 1 performed similarly. These formulas, incorporating TR peak velocity or peak pressure gradient, with or without the addition of estimated RAP, demonstrated a very strong correlation coefficient.

Concerning DPAP, formula 1 in Table 1 exhibited a strong correlation with invasively measured DPAP. Interestingly, including estimated RAP values in the EDPAP1 resulted in a marginal decline in the correlation coefficient. This finding suggests that further refinement may be needed in the echocardiographic methods for estimating RAP in children.

For MPAP, EMPAP1, which employs TR peak pressure gradient as a parameter, revealed a robust correlation coefficient when compared with invasively measured MPAP.

In contrast, none of the 19 dichotomous variables listed in Table 3 demonstrated a significant association with the presence of PH.

Notably, the IVCCI did not exhibit a significant correlation with MRAP. However, this finding may be attributed to the limited sample size, given that invasively measured MRAP data were unavailable for the entire cohort of 55 children.

When compared to the established formulas, the novel formulas exhibited a marginal improvement in the correlation coefficient for DPAP (r = 0.70 vs. r = 0.61) and MPAP (r = 0.80 vs. r = 0.79). However, they demonstrated an identical correlation coefficient for SPAP (r = 0.78). Additionally, for the first time, we introduced a formula for estimating MRAP, which yielded a correlation coefficient of 0.7.

### "Velocity and pressure gradients" variables versus "time" variables: which is preferred for echocardiographic estimation of pulmonary arterial pressure in children with CHD?

This study disclosed that formulas incorporating "velocities and pressure gradients" manifest the strongest correlation with invasively measured values in children, similar to adults. Conversely, most formulas that include "time" parameters, such as the right ventricular outflow tract or PAAT, failed to correlate significantly with the invasively measured values. This observation could be attributed to the greater congruence in pressure gradients across the tricuspid valve between children and adults, compared to the characteristically higher heart rates observed in infants and children. The use of PAAT in predicting PH is contentious. Dammassa et al. [36] analyzed 236 critically ill patients in the intensive care unit and concluded that PAAT is unreliable for estimating pulmonary arterial systolic pressure.

Furthermore, in a study involving 42 children younger than 3 years old, Tai et al. [22] found that heart rate is one of the significant factors affecting PAAT in children. The dependence of PAAT on right ventricular function is also a limitation [36]. On the other hand, Levy et al. [8] reported very high sensitivity and specificity for PAAT < 90 ms and PAAT/RVET of < 0.31 for predicting PH in children. Three primary distinctions existed between our study and the research by Levy et al. [8]. Firstly, Levy et al. [8] employed the older criterion to define PH (MPAP > 25 mmHg), while in contrast, we utilized the updated consensus (MPAP > 20 mmHg). Secondly, in their study, only 13% of patients exhibited a left-to-right shunt, whereas in our study, this condition was present in 84% of patients. Thirdly, they encompassed a heterogeneous patient population, whereas we adhered to the STROBE (Strengthening the Reporting of Observational studies in Epidemiology) guidelines [37]. We made substantial efforts to ensure the homogeneity of our study population to mitigate the potential bias arising from confounding factors. In the same way, Habash et al. [38] found that a PAAT/RVET of less than 0.29 could diagnose PH in patients with 100% sensitivity. The study by Habash et al. [38] was a case–control study on adults who were candidates for liver transplantation.

### Use of dichotomous variables for prediction of pediatric PH

The absence of a statistically significant correlation between any of the dichotomous parameters and the presence of PH might suggest that the age-specific normal range exhibits greater variation within the younger age group. Therefore, defining these parameters based on the normal ranges specific to each age group within the pediatric population is recommended.

### Relationship between IVC dimension and MRAP in infants and children: the importance of BSA-indexed minimal dimension of IVC

In this study, the best correlation was found between invasively measured MRAP and the minimal IVC diameter indexed for BSA. In a study of IVC diameters in 120 normal children aged one to 18 years, Kutty et al. [39] also emphasized the necessity of indexing for BSA. Moreover, without an IVC with a maximum diameter of 2.1 cm or more, the MRAP of 20 mmHg occurred in case number 29 of our study population (see Supplementary Fig. 1 in Additional File 1).

No significant correlation was found between the maximal IVC diameter (nonindexed for BSA) and invasively measured MRAP. Extrapolating the relationship between IVC size, diameter change with inspiration, and MRAP from adults to the pediatric population assumes similarity in vascular dynamics of large veins, including distensibility, compliance, cross-sectional compliance, pressure-strain elastic modulus (Peterson modulus), and Young elastic modulus. However, no studies have been conducted to investigate this assumption thus far [40–42]. The greater distensibility of large veins in infants and children, compared to adults, may explain why using the maximal size of the IVC as a substitute for MRAP can be misleading. Standardization of measurement is crucial, as de Souza et al. [43] demonstrated notable variations in measurements between M-mode and B-mode echocardiography. Furthermore, IVC dilation can occur because of increased IVC capacitance rather than increased pressure, as in certain patients with syncope, as a physiologic adaptation in athletes, or simply because of consuming large quantities of fluids [44–46].

Garcia et al. [47] found that an IVCCI of ≤ 0.24 accurately detects central venous pressure ≥ 10 mmHg. However, none of our cases, including the one with a MRAP of 20 mmHg, had an IVCCI of ≤ 0.24. They studied 70 pediatric patients undergoing cardiopulmonary bypass surgery for CHD. They inserted the catheter in various positions, including the internal jugular vein, peripherally inserted central catheter, femoral vein, and intracardiac line. We did not find a significant relationship between the IVCCI and MRAP, possibly due to differences in study methods or population characteristics (diagnosis, age, sample size). Comparing IVC distensibility in infants, children, and adults is crucial before applying adult guidelines for pediatric RAP estimation based on IVC size and diameter changes.

This study has several limitations. Our study did not include children with cyanotic CHD or patients with arrhythmia. This exclusion was a deliberate choice to ensure our study population's homogeneity. By focusing on a specific subset of patients with noncyanotic CHD, we aimed to reduce variability and eliminate the potential influence of disease-specific factors on our results. While this decision enhances the internal validity of our findings within the selected population, it limits our results' generalizability to the broader spectrum of CHDs. Another limitation of our study was the absence of data on MRAP measured during cardiac catheterization for all the patients.

## Conclusions

This study applied existing formulas and parameters, originally designed for estimating or predicting PH in adults, to a cohort of 55 pediatric patients with acyanotic CHD. The analysis revealed that formulas incorporating tricuspid and pulmonary valve velocities and pressure gradient parameters demonstrated the strongest correlation with invasively measured values in children. Moreover, we introduced robust new equations for estimating SPAP, DPAP, and MPAP, as well as MRAP. These equations utilize three specific echocardiographic parameters: Doppler measurements of TR and PR, in conjunction with the minimum diameter of the IVC.

### Supplementary Information

Additional file 1: Supplementary Tables and Figure.

Additional file 2: Supplementary Material 1.

## Data Availability

The data that support the findings of this study are available from the corresponding author, upon reasonable request.

## Abbreviations

ASE: American Society of Echocardiography
AUC: Area under the curve
BSA: Body surface area
CHD: Congenital heart disease
CI: Confidence interval
DPAP: Diastolic pulmonary arterial pressure
EDPAP: Estimated diastolic pulmonary arterial pressure
EMPAP: Estimated mean pulmonary arterial pressure
ESPAP: Estimated systolic pulmonary arterial pressure
IVC: Inferior vena cava
IVCCI: Inferior vena cava collapsibility index
MPAP: Mean pulmonary arterial pressure
MRAP: Mean right atrial pressure
PAAT: Pulmonary artery acceleration time
PH: Pulmonary hypertension
PR: Pulmonary regurgitation
RAP: Right atrial pressure
RVET: Right ventricular ejection time
SPAP: Systolic pulmonary arterial pressure
STROBE: Strengthening the Reporting of Observational studies in Epidemiology
TR: Tricuspid regurgitation
