# Supplementary material for: Echocardiographic estimation of pulmonary arterial and right atrial pressures in children with congenital heart disease: a comprehensive prospective study and introduction of novel equations
Source: J Cardiovasc Imaging. 2024 Aug 8;32:23. doi: 10.1186/s44348-024-00023-4 (PMC11308456; doi:10.1186/s44348-024-00023-4)
Supplement: Supplementary file 1 — Additional file 1: Supplementary Tables and Figure. [file 44348_2024_23_MOESM1_ESM.docx]

| Supplementary Table 1. Measured echocardiographic parameters | |
| --- | --- |
| 1 | Inferior vena cava minimal diameter (centimeters) |
| 2 | Inferior vena cava maximal diameter (centimeters) |
| 3 | Tricuspid regurgitation peak pressure gradient (mmHg) |
| 4 | Tricuspid regurgitation mean pressure gradient (mmHg) |
| 5 | Tricuspid regurgitation peak velocity (m/sec) |
| 6 | Tricuspid regurgitation velocity time integral (centimeters) |
| 7 | Pulmonary regurgitation peak pressure gradient (mmHg) |
| 8 | Pulmonary regurgitation peak velocity (m/sec) |
| 9 | Pulmonary regurgitation end-velocity (m/sec) |
| 10 | Pulmonary artery acceleration time (milliseconds) |
| 11 | Aortic acceleration time (milliseconds) |
| 12 | Pulmonary artery deceleration time (milliseconds) |
| 13 | Right ventricular isovolumic relaxation time (milliseconds) |
| 14 | Right ventricular isovolumic contraction time (milliseconds) |
| 15 | Right ventricular ejection time (milliseconds) |
| 16 | Right ventricular outflow tract acceleration time (milliseconds) |
| 17 | Tricuspid annular plane systolic excursion (TAPSE) (millimeters) |
| 18 | Tricuspid Sm velocity (cm/sec) |
| 19 | Tricuspid Sm velocity time integral (centimeters) |
| 20 | Tricuspid valve E velocity (cm/sec) |
| 21 | Tricuspid valve Em velocity (cm/sec) |
| 22 | Mitral valve E velocity (cm/sec) |
| 23 | Mitral valve Em velocity (cm/sec) |
| 24 | Basal right ventricular dimension (centimeters) |
| 25 | Mid-right ventricular diameter (centimeters) |
| 26 | Right ventricular length (centimeters) |
| 27 | Left ventricular dimension (millimeters) |
| 28 | Pulmonary artery dimension (centimeters) |
| 29 | Aortic dimension (millimeters) |

| Supplementary Table 2. Association between predictive dichotomous variables for identifying pulmonary hypertension and invasively measured mean pulmonary arterial pressure in 55 children with congenital heart disease | | | |
| --- | --- | --- | --- |
|  | **Parameter** | **Pearson Chi-square**  ***P* value**  **(two-sided)** | **Fisher's Exact test**  ***P* value**  **( two-sided)** |
| 1 | Tricuspid Sm velocity < 12 cm/seconds (sec) | 0.678 | - |
| 2 | Right ventricular isovolumic relaxation time (IVRT)> 75milliseconds (ms) | - | 1 |
| 3 | Acceleration time of right ventricular outflow tract < 100 ms | - | 0.166 |
| 4 | Pulmonary artery acceleration time < 90 ms | 0.118 | - |
| 5 | Pulmonary artery acceleration time <60 ms | - | 1 |
| 6 | Pulmonary artery acceleration time /right ventricular ejection time < 0.31 | 0.276 | - |
| 7 | Pulmonary artery acceleration time /right ventricular ejection time < 0.29 | - | 0.188 |
| 8 | Pulmonary artery acceleration time /right ventricular ejection time < 0.25 | - | 0.210 |
| 9 | Tricuspid annular plane systolic excursion (TAPSE) < 16 mm | 0.393 | - |
| 10 | Tei index> 0.36 | 0.877 | - |
| 11 | Tei index> 0.80 | - | 0.257 |
| 12 | The ratio of RV basal diameter /LV basal diameter >1 | 0.023 | - |
| 13 | Pulmonary artery/ aortic size > 1.5 | - | 0.257 |
| 14 | Pulmonary artery/ aortic size > 2 | - | 1 |
| 15 | Pulmonary artery acceleration time / aortic acceleration time ≤1 | 0.351 | - |
| 16 | Pulmonary artery acceleration time / aortic acceleration time ≤ 0. 7 | - | 0.504 |
| 17 | Mid-systolic notch in the Doppler of right ventricular outflow tract | 0.315 | - |
| 18 | Pulmonary artery acceleration time/ Right ventricular ejection time <0.23 | - | 0.104 |
| 19 | Pulmonary artery acceleration time / Pulmonary artery deceleration time < 0.3 | - | 0.061 |


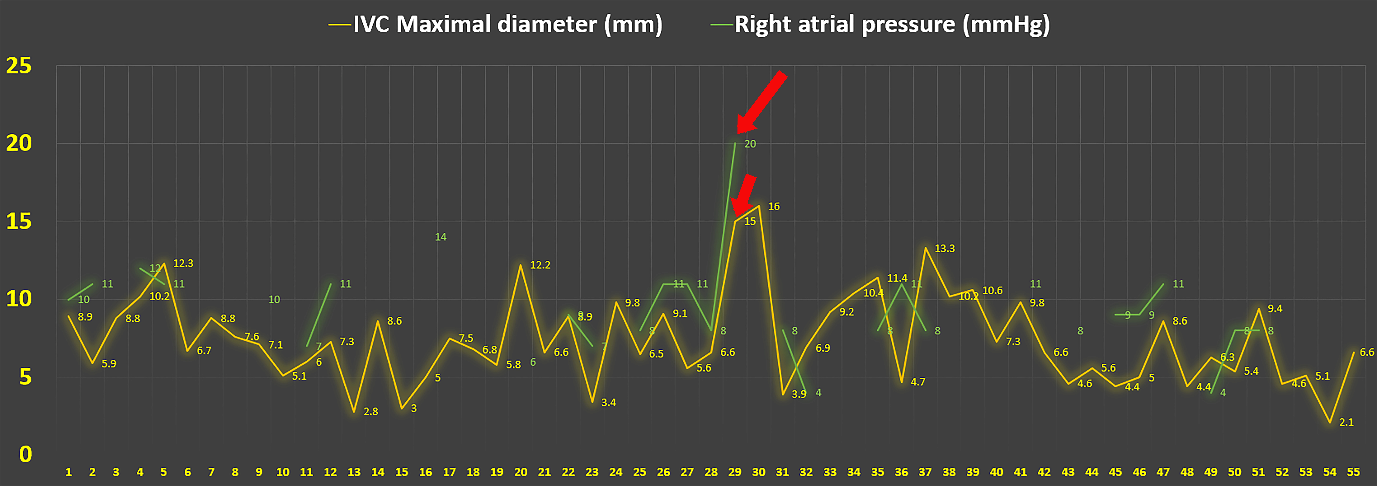


**Supplementary Fig. 1.** The X-axis shows the case numbers. The yellow curve indicates the inferior vena cava diameter in millimeters, and the green line depicts the mean right atrial pressure measured at cardiac catheterization. Gaps indicate missing data.
