# Supplementary material for: Echocardiographic estimation of pulmonary arterial and right atrial pressures in children with congenital heart disease: a comprehensive prospective study and introduction of novel equations
Source: J Cardiovasc Imaging. 2024 Aug 8;32:23. doi: 10.1186/s44348-024-00023-4 (PMC11308456; doi:10.1186/s44348-024-00023-4)
Supplement: Supplementary file 2 — Additional file 2: Supplementary Material 1. [file 44348_2024_23_MOESM2_ESM.docx]

**Supplementary Material 1**

- In the following cross-tabulations, cells representing true positives and true negatives are highlighted in light green.

| **Table 1.** Cross-tabulation of the relationship between tricuspid s-wave velocity and pulmonary hypertension | | | | |
| --- | --- | --- | --- | --- |
|  | | Tricuspid Sm velocity (centimeters/second) | | Total |
|  |  | <12 centimeters/second | ≥12 centimeters/second |  |
| Invasively measured mean pulmonary arterial pressure | Normal | 6 | 17 | 23 |
|  | PH | 10 | 22 | 32 |
| Total | | 16 | 39 | 55 |

| **Table 2.** Cross-tabulation of the relationship between right ventricular isovolumic relaxation time and pulmonary hypertension | | | | |
| --- | --- | --- | --- | --- |
|  | | Right ventricular isovolumic relaxation time (milliseconds) | | Total |
|  |  | ≤75 milliseconds | >75 milliseconds |  |
| Invasively measured mean pulmonary arterial pressure | Normal | 22 | 1 | 23 |
|  | PH | 30 | 2 | 32 |
| Total | | 52 | 3 | 55 |

| **Table 3.** Cross-tabulation of the relationship between right ventricular outflow tract acceleration time and pulmonary hypertension | | | | |
| --- | --- | --- | --- | --- |
|  | | Right ventricular isovolumic relaxation time | | Total |
|  |  | < 100 milliseconds | ≥ 100 milliseconds |  |
| Invasively measured mean pulmonary arterial pressure | Normal | 2 | 21 | 23 |
|  | PH | 8 | 24 | 32 |
| Total | | 10 | 45 | 55 |

| **Table 4.** Cross-tabulation of the relationship between the presence of the mid-systolic notch and pulmonary hypertension | | | | |
| --- | --- | --- | --- | --- |
|  | | Mid-systolic notch | | Total |
|  |  | Absent | Present |  |
| Invasively measured mean pulmonary arterial pressure | Normal | 12 | 11 | 23 |
|  | PH | 21 | 11 | 32 |
| Total | | 33 | 22 | 55 |

| **Table 5.** Cross-tabulation of the relationship between RV/ LV basal diameter > 1 and pulmonary hypertension | | | | |
| --- | --- | --- | --- | --- |
|  | | RV/ LV basal diameter | | Total |
|  |  | RV/ LV basal diameter ≤ 1 | RV/ LV basal diameter > 1 |  |
| Invasively measured mean pulmonary arterial pressure | Normal | 20 | 2 | 22 |
|  | PH | 19 | 9 | 28 |
| Total | | 39 | 11 | 50 |

| **Table 6.** Cross-tabulation of the relationship between pulmonary artery acceleration time < 90 milliseconds and pulmonary hypertension | | | | |
| --- | --- | --- | --- | --- |
|  | | Pulmonary artery acceleration time | | Total |
|  |  | < 90 milliseconds | ≥ 90 milliseconds |  |
| Invasively measured mean pulmonary arterial pressure | Normal | 0 | 23 | 23 |
|  | PH | 1 | 31 | 32 |
| Total | | 1 | 54 | 55 |

| **Table 7.** Cross-tabulation of the relationship between pulmonary artery acceleration time < 60 milliseconds and pulmonary hypertension | | | | |
| --- | --- | --- | --- | --- |
|  | | Pulmonary artery acceleration time | | Total |
|  |  | < 60 milliseconds | ≥ 60 milliseconds |  |
| Invasively measured mean pulmonary arterial pressure | Normal | 0 | 23 | 23 |
|  | PH | 1 | 31 | 32 |
| Total | | 1 | 54 | 55 |

| **Table 8.** Cross-tabulation of the relationship between pulmonary artery acceleration time/right ventricular ejection time < 0.31 and pulmonary hypertension | | | | |
| --- | --- | --- | --- | --- |
|  | | Pulmonary artery acceleration time/right ventricular ejection time | | Total |
|  |  | <0.31 | ≥0.31 |  |
| Invasively measured mean pulmonary arterial pressure | Normal | 5 | 18 | 23 |
|  | PH | 11 | 18 | 29 |
| Total | | 16 | 36 | 52 |

| **Table 9.** Cross-tabulation of the relationship between pulmonary artery acceleration time/right ventricular ejection time < 0.29 and pulmonary hypertension | | | | |
| --- | --- | --- | --- | --- |
|  | | Pulmonary artery acceleration time/right ventricular ejection time | | Total |
|  |  | <0.29 | ≥0.29 |  |
| Invasively measured mean pulmonary arterial pressure | Normal | 4 | 19 | 23 |
|  | PH | 10 | 19 | 29 |
| Total | | 14 | 38 | 52 |

| **Table 10.** Cross-tabulation of the relationship between pulmonary artery acceleration time/right ventricular ejection time < 0.25 and pulmonary hypertension | | | | |
| --- | --- | --- | --- | --- |
|  | | Pulmonary artery acceleration time/right ventricular ejection time | | Total |
|  |  | <0.25 | ≥0.25 |  |
| Invasively measured mean pulmonary arterial pressure | Normal | 1 | 22 | 23 |
|  | PH | 5 | 24 | 29 |
| Total | | 6 | 46 | 52 |

| **Table 11.** Cross-tabulation of the relationship between pulmonary artery acceleration time/right ventricular ejection time < 0.23 and pulmonary hypertension | | | | |
| --- | --- | --- | --- | --- |
|  | | Pulmonary artery acceleration time/right ventricular ejection time | | Total |
|  |  | <0.23 | ≥0.23 |  |
| Invasively measured mean pulmonary arterial pressure | Normal | 0 | 23 | 23 |
|  | PH | 3 | 25 | 28 |
| Total | | 3 | 48 | 51 |

| **Table 12.** Cross-tabulation of the relationship between tricuspid annular plane excursion (TAPSE) less than 16 mm and pulmonary hypertension | | | | |
| --- | --- | --- | --- | --- |
|  | | Tricuspid annular plane excursion (TAPSE) | | Total |
|  |  | <16 mm | ≥16 mm |  |
| Invasively measured mean pulmonary arterial pressure | Normal | 5 | 18 | 23 |
|  | PH | 10 | 21 | 31 |
| Total | | 15 | 39 | 54 |

| **Table 13.** Cross-tabulation of the relationship between aTei index of more than 0.36 and pulmonary hypertension | | | | |
| --- | --- | --- | --- | --- |
|  | | Tei index | | Total |
|  |  | ≤ 0.36 | >0.36 |  |
| Invasively measured mean pulmonary arterial pressure | Normal | 11 | 12 | 23 |
|  | PH | 14 | 15 | 29 |
| Total | | 25 | 27 | 52 |

| **Table 14.** Cross-tabulation of the relationship between a Tei index of more than 0.80 and pulmonary hypertension | | | | |
| --- | --- | --- | --- | --- |
|  | | Tei index | | Total |
|  |  | ≤ 0.80 | >0.80 |  |
| Invasively measured mean pulmonary arterial pressure | Normal | 23 | 0 | 23 |
|  | PH | 28 | 1 | 29 |
| Total | | 51 | 1 | 52 |

| **Table 15.** Cross-tabulation of the relationship between pulmonary artery/ aortic size and pulmonary hypertension | | | | |
| --- | --- | --- | --- | --- |
|  | | pulmonary artery/ aortic size | | Total |
|  |  | ≤ 1.5 | >1.5 |  |
| Invasively measured mean pulmonary arterial pressure | Normal | 23 | 0 | 23 |
|  | PH | 29 | 3 | 32 |
| Total | | 52 | 3 | 55 |

| **Table 16.** Cross-tabulation of the relationship between pulmonary artery/ aortic size and pulmonary hypertension | | | | |
| --- | --- | --- | --- | --- |
|  | | pulmonary artery/ aortic size | | Total |
|  |  | ≤ 2 | >2 |  |
| Invasively measured mean pulmonary arterial pressure | Normal | 23 | 0 | 23 |
|  | PH | 31 | 1 | 32 |
| Total | | 54 | 1 | 55 |

| **Table 17.** Cross-tabulation of the relationship between pulmonary artery acceleration time / pulmonary artery deceleration time < 0.3 and pulmonary hypertension | | | | |
| --- | --- | --- | --- | --- |
|  | | pulmonary artery acceleration time / pulmonary artery deceleration time | | Total |
|  |  | <0.3 | ≥ 0.3 |  |
| Invasively measured mean pulmonary arterial pressure | Normal | 0 | 23 | 23 |
|  | PH | 4 | 25 | 29 |
| Total | | 4 | 48 | 52 |

| **Table 18.** Cross-tabulation of the relationship between pulmonary artery acceleration time / aortic acceleration time ≤ 1 and pulmonary hypertension | | | | |
| --- | --- | --- | --- | --- |
|  | | pulmonary artery acceleration time / aortic acceleration time | | Total |
|  |  | ≤1 | >1 |  |
| Invasively measured mean pulmonary arterial pressure | Normal | 4 | 19 | 23 |
|  | PH | 9 | 23 | 32 |
| Total | | 13 | 42 | 55 |

| **Table 19.** Cross-tabulation of the relationship between pulmonary artery acceleration time / aortic acceleration time ≤ 0.7 and pulmonary hypertension | | | | |
| --- | --- | --- | --- | --- |
|  | | pulmonary artery acceleration time / aortic acceleration time | | Total |
|  |  | ≤0.7 | >0.7 |  |
| Invasively measured mean pulmonary arterial pressure | Normal | 0 | 23 | 23 |
|  | PH | 2 | 30 | 32 |
| Total | | 2 | 53 | 55 |
